# RAB11-Mediated Trafficking and Human Cancers: An Updated Review

**DOI:** 10.3390/biology10010026

**Published:** 2021-01-04

**Authors:** Elsi Ferro, Carla Bosia, Carlo C. Campa

**Affiliations:** 1 Department of Applied Science and Technology, Politecnico di Torino, 24 Corso Duca degli Abruzzi, 10129 Turin, Italy; elsi.ferro@polito.it (E.F.); carla.bosia@polito.it (C.B.); 2 Italian Institute for Genomic Medicine, c/o IRCCS, Str. Prov. le 142, km 3.95, 10060 Candiolo, Italy

**Keywords:** RAB11, RAB25, endosome, sorting, cancer

## Abstract

**Simple Summary:**

The small GTPase RAB11 is a master regulator of both vesicular trafficking and membrane dynamic defining the surface proteome of cellular membranes. As a consequence, the alteration of RAB11 activity induces changes in both the sensory and the transduction apparatuses of cancer cells leading to tumor progression and invasion. Here, we show that this strictly depends on RAB11′s ability to control the sorting of signaling receptors from endosomes. Therefore, RAB11 is a potential therapeutic target over which to develop future therapies aimed at dampening the acquisition of aggressive traits by cancer cells.

**Abstract:**

Many disorders block and subvert basic cellular processes in order to boost their progression. One protein family that is prone to be altered in human cancers is the small GTPase RAB11 family, the master regulator of vesicular trafficking. RAB11 isoforms function as membrane organizers connecting the transport of cargoes towards the plasma membrane with the assembly of autophagic precursors and the generation of cellular protrusions. These processes dramatically impact normal cell physiology and their alteration significantly affects the survival, progression and metastatization as well as the accumulation of toxic materials of cancer cells. In this review, we discuss biological mechanisms ensuring cargo recognition and sorting through a RAB11-dependent pathway, a prerequisite to understand the effect of RAB11 alterations in human cancers.

## 1. Introduction

To manage pathogenic alterations induced by genetic/epigenetic/transcriptomic changes, cells evolved various strategies to exploit cellular machinery and guarantee their own survival. Specific genetic alterations modify key elements controlling the molecular composition of the cell surface, the assembly of internal organelles and the efficacy of intracellular and extracellular transmission. RAB GTPases are key players in membrane transport events and are fundamental to ensure the efficacy of intracellular logistics [1,2,3].

RAB GTPases are members of the Ras superfamily of monomeric G proteins, a large protein family which includes among its members RAS, RHO–RAC, ARF and RAN as well as the more recently characterized RHEB, RAD and RIT subfamilies [4]. Similarly to other small GTPases, Rab proteins are molecular switches that cycle between an active and an inactive state through the association and the subsequent hydrolysis of guanosine-5′-triphosphate (GTP). In particular, association with GTP is stimulated by Guanine nucleotide exchange factors (GEFs), whereas hydrolysis of GTP to GDP is promoted by GTPase-activating proteins (GAPs) [5]. This activation cycle is pivotal for the binding of multiple effector proteins which mediate the delivery of cellular material to various intracellular compartments such as—the plasma membrane, endosomes, autophagosomes, leading edge of migrating cells, midbodies, primary cilium and centrioles [6]. As a matter of fact, alterations of RAB-mediated trafficking are pivotal in various aspects of both progression and tumorgenicity of cancer cells including the sustainment of proliferative signaling, the evasion of growth suppression, the induction of receptor recycling, the activation of invasion and metastasis as well as the reprogramming of tumor metabolism and the evasion of immune destruction [7,8].

## 2. Different RAB11 Isoforms for Distinct Cellular Functions

**Figure 1:**
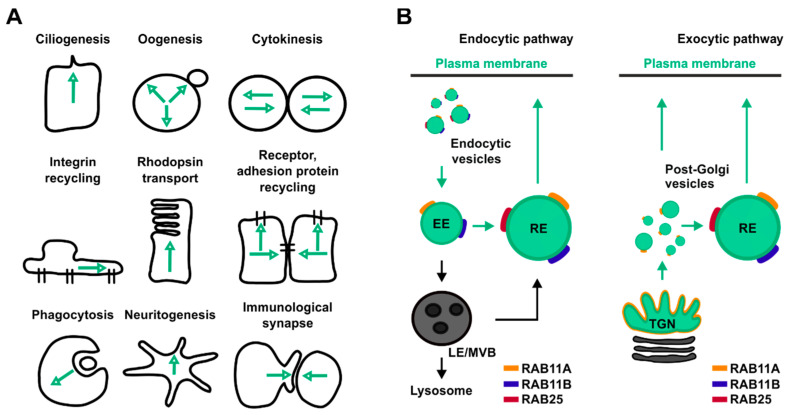
Overview of the main cellular processes and trafficking pathways regulated by RAB11 isoforms. (**A**) Schematic representation of cellular processes controlled by RAB11. Black lines represent cells’ contour, green arrows represent directions of RAB11-mediated trafficking pathways. (**B**) Schematic representation of RAB11 isoform localization in both endocytic (left panel) and exocytic pathways (right panel). Green circles represent RAB11-positive compartments, yellow, blue, red lines represent RAB11A, RAB11B and RAB25, respectively.

One prominent Rab-mediated transport pathway that cancer cells exploit to adapt their internal states to fluctuations of both their gene expression and microenvironmental status is the RAB11-mediated trafficking pathway. This transport route relies on members of the RAB11 protein family which control the delivery of both proteins and lipids toward several organelles and therefore are involved in many cellular processes (Figure 1A,B).

The RAB11 protein family includes three different isoforms named RAB11A, RAB11B and RAB25, each one encoded by a different gene located in a distinct chromosome (Table 1). All RAB11 isoforms are widely distributed in human tissues. In particular, while RAB11A is ubiquitously expressed, both RAB11B and RAB25 are enriched in specific organs (Table 1) [9]. Notably, RAB11 proteins differ in their percentage of sequence identity. While RAB11A and RAB11B share 89% of sequence similarity, the identity between RAB25 with either RAB11A or RAB11B is lower than 70% (Table 1) [10].

Although minimal, such differences in protein sequence identity significantly impact both protein structure and specificity of effector binding, providing an explanation for the different roles of RAB11 isoforms in cells [11,12]. All RAB11 proteins present several post-translational modifications, including prenylation, phosphorylation and ubiquitination. In particular, while prenylation at RAB11 C-termini allows Rab membrane association, both phosphorylation and ubiquitination modulate either activity or degradation of RAB11 isoforms, respectively [9].

RAB11A is the first discovered and best characterized member of the RAB11 subfamily. Homozygous depletion of *Rab11a* in mice is embryo lethal; whereas the loss of RAB11A protein in neurons does not significantly impact both brain development and functionality. In contrast, depletion of *Rab11a* in mice intestine increases intracellular accumulation of apical proteins and it induces shortening of microvilli [13,14].

In cells, RAB11A localizes to the recycling endosome (RE) a perinuclear positioned membrane-bound compartment implicated in the control of both proteins and lipids trafficking through and from the RE to the plasma membrane (Figure 1B) [15,16]. In addition, RAB11A mediates transport of vesicular cargoes from peripheral sorting endosomes (SEs) to the RE (Figure 1A) [17]. Furthermore, RAB11A localizes at the trans-Golgi network (TGN), where it controls the transport of material towards both the apical and the basolateral membrane in polarized cells (Figure 1B) [18]. Based on both its wide distribution and functions aimed at mediating the delivery of cellular material to both plasma membrane, endosomes, autophagosome and midbody, RAB11A influences several cellular processes such as cytokinesis, phagocytosis, cell migration and proliferation, immunological synapse assembly, ciliogenesis, influenza virus replication, and autophagy [19,20,21,22,23,24,25].

The RAB11B isoform is highly enriched in the brain and, consistently, homozygous depletion of *Rab11b* in mice leads to neurological dysfunction (data from International Mouse Phenotyping Consortium, https://www.mousephenotype.org/). Although, RAB11B localizes in both the RE and SE similarly to RAB11A, RAB11B depletion does not alter accumulation of the Transferrin receptor (TfR) at the perinuclear RE whereas downregulation of RAB11A does, thereby indicating a non-overlapping function for these small GTPases in endocytic recycling [26]. In parallel, accumulation of fibroblast growth factor receptor 4 (FGFR4) was observed after downregulation of RAB11B, but not RAB11A [27]. Consistently, RAB11B localizes to an apical pericentrosomal membrane-bound compartment distinct from RAB11A [28]. In this context, recent findings indicate that RAB11A and RAB11B perform opposing roles in the same cells. In particular, RAB11B was found to control recycling of the PAR1 receptor from the endosome to plasma membrane while RAB11A defines the rate of PAR1 degradation by delivering such signaling receptor into the autophagic pathway [29]. Lastly, in polarized epithelial cells, RAB11B specifically mediates the apical transport of the cystic fibrosis transmembrane conductance regulator (CFTR) [30] while it regulates exocytosis in neurons and neuroendocrine cells [31].

RAB25-deficient mice do not exhibit gross pathology [32]. However, when *Rab25* depletion is associated with a specific genetic background, as found in 129/J mouse strain, it induces vaginal cancer in virgin females and distal esophageal stricture in both males and females [32]. The localization of RAB25 in cells partially overlap with those of RAB11A (Figure 1B) [33], whereas its role has been associated both in transport of oncogenic signaling receptors and in cellular bioenergetics [9,34].

Through both the presence of various isoforms, the wide distribution amongst distinct cellular compartments and the occurrence of several post-translational mechanisms of regulation, they confer to RAB11 and its trafficking route an elevated level of plasticity. Consequently, RAB11-mediated trafficking pathways provide essential support to various aspects of cellular processes.

In the sections below, we will discuss the implications of RAB11′s role in the planning, implementation and control of the efficient and effective flow and storage of cellular materials, with the aim at understanding the basic principles governing cellular processes underlying tumor progression.

## 3. RAB11-Mediated Trafficking Sustains Proliferative Signaling

One of the fundamental traits of cancer cells is their ability to sustain chronic proliferation. Growth-promoting signals are tightly regulated in normal cells and they are exploited to instruct cells about both their microenvironment, their duties and their fate. As a consequence, both growth factors, signaling receptors and intracellular signaling cascades are essential to ensure normal tissue homeostasis. Therefore, it is not surprising that by deregulating these signals, cancer cells become independent of extracellular messages that maintain normal tissue homeostasis and thus turn into masters of their own destiny [35].

Typically, binding of extracellular growth factors to surface receptors such as tyrosine kinases provides the key event that induces activation of proliferative signaling cascades in cells. Cancer cells acquire the capability to sustain such a mitogenic response in a number of alternative ways such as inducing autocrine signaling, increasing the number of mitogenic receptors on the cell surface, or extending the mitogenic stimulation by altering the information processing of downstream signaling cascades [36]. RAB11 isoforms were described to influence these processes by controlling the intracellular distribution of cargoes such as growth factor receptors, transmembrane receptors, cell adhesion molecules and signaling lipids [36]. The function of RAB11 isoforms in the cellular logistic implies a role for this small GTPase family in the organization and implementation of operations ensuring the flow of information between distinct cellular districts with spatiotemporal precision. Such procedures direct both the generation, packaging, transportation and stocking of membrane cargoes at the right place and at the right time [5,37]. Therefore, it is conceivable that RAB11 isoforms serve multiple roles impacting various cellular processes when dysfunctional. However, it remains still difficult to predict RAB11 functions in human diseases.

This is exemplified by the dual character of RAB11 isoforms in human cancers. RAB11 isoforms both enhance and diminish cancer progression by functioning either as oncogenes or as tumor suppressors (Table 2, Table 3 and Table 4). 

Loss of RAB11 isoforms (i.e., RAB11A and RAB25) is not tumorigenic per se and results in both hyperplasia and dysplasia of colorectal tissues [32,60], whereas their overexpression is not sufficient to transform nontumorigenic/immortalized cells, rather they promote the acquisition of both metastatic and invasive properties in already transformed cells [34,43,51]. Conversely, in cells characterized by a compromised genetic background, alterations in the expression of RAB11 isoforms promote cancer progression. Notably, this occurs both when RAB11 isoforms are downregulated and when they are overexpressed (Table 2, Table 3 and Table 4). As an example, in epithelial cancer cells characterized by loss of either APC or SMAD3 proteins, RAB11 isoforms display a tumor suppressor function promoting both proliferation and aggressiveness of cancer cells when downregulated [32,61]. On the contrary, RAB11 family members show oncogenic potential by promoting proliferation and invasiveness cancer cells when overexpressed [51,62].

Notably, recent evidence suggests that alteration of RAB11 gene expression is part of the adaptive programs that cells exploit to match environmental changes. In particular, RAB11B is up-regulated during adaptation of breast cancer cells to the brain microenvironment, a process that induces the massive modification of the cell surface proteome [43]. Intriguingly, the finding that, in breast cancer, other RAB11 isoforms (i.e., RAB25) are either not altered or epigenetically silenced, strongly support the idea that RAB11 proteins perform non-overlapping functions in cancer [43,63,64].

Despite the fact that these oncogenic phenotypes have been mechanistically linked to an alteration in both proliferative and migratory cellular capacity through either recycling of receptor tyrosine kinases, E-Cadherin, α5β1 Integrins, or to activation of oncogenic signaling and metabolic stress, the molecular underpinnings of these tumorigenic events are still poorly understood [33,34,51,65,66,67,68]. However, we are still struggling to understand how RAB11-mediated trafficking is orchestrated, the evidence that RAB11 acts in concert with an elevated number of effectors and interactors, to ensure the fidelity of intracellular trafficking, provides a novel framework that allows to interpret RAB11-induced cellular dysfunction.

## 4. RAB11-Mediated Sorting Controls Intracellular Accumulation of Surface Proteins

Most growth factor receptors and transmembrane proteins are transported to the cell surface by RAB11 isoforms. This event confers excitability to mitogenic stimulation. By deregulating this transport route, cancer cells increase the number of growth factor receptors on the plasma membrane prompting the amplification of mitogenic stimulation. 

An elevated number of reports confirms the significance of the RAB11-mediated trafficking pathway in transport of pro-tumorigenic signaling receptors to cell surface. In particular, RAB11A as well as RAB11B and RAB25 enhance receptor recycling to the cell surface, and, they concomitantly decrease degradation in cytoplasm [12,43,61]. As a consequence, RAB11-mediated trafficking supports both the amplification and duration of signaling cascades by increasing receptor half-life in the plasma membrane. In addition, the directional flux of membrane cargoes from internal organelles towards the cell surface promotes directional cell migration of cancer cells and therefore their ability to invade healthy tissue and metastasize to distant sites. Accordingly, RAB11 proteins were found to recycle β1-Integrin to the plasma membrane [32,43] and in parallel their depletion blocks receptors inside cells for a longer time, with major consequences in terms of amplification of oncogenic signaling cascades [43,69,70].

However, the evidence that RAB11 isoforms function both as oncogenes and tumor suppressors challenges this view, raising the possibility that in some cellular contexts, such as in the presence of massive alteration in both signaling and metabolic pathways, RAB11 might misdirect cargoes assigned to the cell surface to a different destination. 

In this context, the analysis of both specific and overlapping RAB11 roles might provide novel insights into mechanisms controlling intracellular accumulation of surface proteins. As an example, RAB11A and RAB11B perform divergent roles in the regulation of PAR1 receptor trafficking by controlling either its degradation or its recycling, respectively. In the absence of RAB11B-mediated recycling, the PAR1 receptor is trafficked to autophagic organelles for degradation, a process that requires RAB11A. Accordingly, RAB11A knockdown restores PAR1 expression in silenced RAB11B cells [29].

## 5. RAB11 Balances Distinct Trafficking Routes with Opposing Functions

Balance between either recycling, intracellular accumulation or degradation of surface receptors is ensured by a vast number of intracellular membrane compartments that both evolve and mature dynamically to connect both the endocytic and the exocytic pathway. In particular, at the crossroads between recycling and degradation routes resides the early endosome, the sorting station of the endocytic system. The role of early endosomes is to accept endocytosed proteins and lipids and to redirect them either back to plasma membrane or to lysosomes. Serving these two intracellular highways, two opposing sorting machineries operate at the early endosome and both require the assembly of multiprotein complexes. In particular, targeted degradation of surface receptors is controlled by the endosomal sorting complex required for transport (ESCRT), while the recycling to the cell surface of endocytosed cargo is ensured by distinct molecular assemblies such as the retromer/SNX27/WASH complex, the Retriever complex, and the FERARI complex [71]. Notably, both retromer/SNX27/WASH complex and Retriever share common regulators such as the Vacuolar protein sorting-associated protein 29 (VPS29) and the WASH complex. In particular, VPS29 is essential for cargo selection, transcytosis of the polymeric immunoglobulin receptor (pIgR-pIgA) and recycling of NxxY-motif-containing cargo proteins by coupling to SNX17 [72,73,74,75], whereas the WASH complex functions as a nucleation-promoting factor (NPF) at the surface of endosomes, where it induces actin polymerization and fission of recycling tubules containing either transferrin receptor (TfR), EGFR or α5β1-Integrin [71,76,77,78,79,80]. Remarkably, despite the similarities between the retromer/SNX27/WASH and Retriever complexes, it is not known whether Rab proteins participate in the endosomal recruitment of these recycling machineries. 

Recently, the FERARI complex has emerged as a critical component in RAB11-mediated trafficking. In particular, it promotes membrane fission and fusion at the recycling compartment of early/sorting endosomes [81]. The FERARI complex consists of three interacting units—the Rab-module, the SNARE module, and the membrane-fission module. These elements coordinate respectively—(i) tethering of RAB11 vesicle to RAB5 positive early endosome, (ii) fusion of incoming RAB11 vesicle and, (iii) fission of the newly generated RAB11 vesicle from the endosome. Notably, these modules act synergistically, thereby coordinating kiss-and-run vesicle release for cargo delivery at the recycling pathways [81].

The important role of the FERARI complex in controlling RAB11-mediated trafficking relies on the Rab-module. This functional unit includes the Rab effectors Rabenosyn-5 (RBSN) and RAB11FIP5, two proteins connecting the endocytic and recycling transport machinery [81]. In particular, the RAB5 effector RBSN controls both membrane fusion and membrane trafficking of recycling endosomes generated at the early endosome and, interestingly, it is considered a tumor-suppressive gene [82]. In parallel, RAB11FIP5 belongs to the RAB11 family interacting protein (RAB11FIP), a protein family that comprises five distinct genes (i.e., RAB11FIP1, RAB11FIP2, RAB11FIP3, RAB11FIP4, RAB11FIP5) which are characterized by both their binding to RAB11 isoforms and their tumorigenic potential when overexpressed [65,83,84,85,86,87,88,89,90]. Although RAB11FIPs are associated with the recycling of pro-tumorigenic receptors, they do not directly bind surface oncogenic proteins even if both molecular species were found in the same macromolecular complexes [65,91]. Therefore, the RAB11-RAB11FIPs interaction has the purpose of linking recycling vesicles to cytoskeletal transport and tethering machineries rather than regulating receptor sorting.

In FERARI, the receptor sorting function is executed by SNX1, a component of the FERARI’s fission module. SNX1 is a member of the sorting nexin (SNX) protein family that includes 33 distinct genes which share the ability to associate with intracellular membranes containing phosphatidylinositol 3-phosphate (PtdIns(3P)), through their lipid-binding PX domain [92,93]. Moreover, recent evidences indicate that SNX’s PX domain interacts with specific aminoacidic motifs present in plasma membrane receptors [94]. In addition, the presence of a BAR domain gives to SNX1 the ability to sense membrane curvature [95]. As a consequence, SNX1 coordinates sequence-dependent cargo recognition with membrane remodeling to generate cargo-enriched tubulo-vesicular transport carriers. Therefore, SNX1 provides an essential element to control both export and transport of numerous cargoes to cell surface. However, despite these biochemical indications, the role of this SNX1 in human cancers remains debated.

The activity of SNX1 in RAB11-mediated trafficking suggests that such sorting nexin functions as an oncogene by increasing the amount of transmembrane proteins at the plasma membrane when overexpressed. In marked contrast, SNX1 targets EGFR to the lysosome for degradation, thereby decreasing the amount of EGFR available for ligand binding at the cell surface when overexpressed [96]. This designates SNX1 as a potential tumor suppressor involved in transport from the early endosome to the lysosomal compartment [96]. Accordingly, SNX1 downmodulation increases receptor accumulation in endosomes thereby amplifying the mitogenic response downstream of EGFR [97]. Although experimental data appear incompatible with one another, as they link SNX1 with transport of membranes towards both the plasma membrane and lysosome, recent reports address this apparent contradiction. 

SNX-1 was found to sort cargo away from degradation. In particular, SNX1 generates specific regions on endosome, termed microdomains, in which the degradative and recycling pathways occupy distinct portions of membrane surface [98]. The depletion of SNX1 mediates cargo degradation by concentrating ubiquitinated cargoes and organizing the activities for its degradation [98]. As a consequence, loss of SNX1 function might result in a decreased endocytic recycling and a parallel accumulation of cargoes into the degradative organelle. The ability of SNX1 to organize trafficking of signaling receptors by controlling the juxtaposition of adjacent microdomains highlights the relevance of sub-compartmental organization of the sorting endosome in membrane transport and, in particular, in RAB11-mediated trafficking. In theory, to preserve the ability of the early endosome to sort cargoes, the two distinct molecular machineries that direct incoming molecules either to the degradative or to the recycling pathway need to be maintained spatially segregated. If one takes over the entire endosome, sorting might fail and cargo will be misdirected [99]. In such a “winner takes all” process, mechanisms which promote the functional association between distinct membrane domains within the same endosome, will provide a novel theoretical framework over which to interpret the impact of genetic alterations in endocytic genes. In this context, it appears to be essential how RAB11 might cross-regulate assembly or disassembly of opposing microdomains.

## 6. RAB11 Sets the Time for PtdIns Conversion on Endosomal Membrane Microdomain

Endocytic Rabs including RAB4, RAB5 and RAB11 were among the first proteins to be recognized as markers of endosomal membrane microdomains [100]. These small GTPases mark, at various degrees, distinct portions of the early endosome’s surface, thereby revealing extensive compartmentalization within the same continuous membrane [19,101]. Such spatial segregation is ensured by the RAB “GTPase switch” which connects the localization of RAB proteins to their biological function with spatiotemporal precision. Therefore, by controlling the RAB activation cycle, distinct complexes with both different and mutually exclusive function can be connected, thus providing a strategy to control the biochemical identity of membrane both over time and across space. In this context, the direct visualization that RAB11 marks tiny regions of early endosome surface where recycling cargoes accumulate hint at a role for the RAB11 “GTPase switch” in endosomal sorting.

Inactivation of RAB11 reduces the number of recycling tubules emerging from the early endosome, thereby providing evidence that RAB11 activity determines the efficacy of receptor transport to the cell surface [17]. Interestingly, RAB11 activation is coupled with generation of PtdIns(3P), the early endosome resident lipid, a process that requires the phosphorylation of the PtdIns inositol moieties by lipid kinase enzymes [102]. In particular, several reports link the activation of RAB11 to the production of PtdIns(3P) by PI3KC2A, a member of the well-known oncogene PI3K [17,103,104]. Notably, PtdIns(3P) activates RAB11 and, as a consequence, the PI3KC2A depletion reduces RAB11-GTP level in cells. This, in turn, decreases the release of recycling vesicles from the early endosome [17].

Although PtdIns(3P) generation and the subsequent RAB11 activation are essential to induce the production of recycling vesicles from early endosomes, the parallel conversion of PtdIns(3P) to PtdIns defines the timing for fission of such recycling carries [17]. As an example, PtdIns(3P) accumulation blocks the recycling of receptors such as β1-integrin [105].

In cells, PtdIns(3P) accumulation necessitates the depletion of PtdIns(3P)-phosphatases, a protein family that is responsible for the conversion of PtdIns(3P) to PtdIns. The depletion of PtdIns(3P)-phosphatases, such as MTM1, increases PtdIns(3P) in endosomes, thereby resulting in delayed transport of RAB11-positive vesicles. Notably, active RAB11 interacts with MTM1 promoting the endosomal localization of MTM1 [17]. This event leads to failure in localizing SNX1 on the early endosome membrane preventing TfR recycling and delaying EGFR trafficking [106].

The functional interplay between RAB11, PtdIns(3P) and components of the endocytic recycling machinery is underlined by recent works on the COMMD/CCDC22/CCDC93 (CCC) complex. The CCC complex is required for both retromer- and retriever-dependent protein trafficking and hence it defines the transport of β1-Integrin, LDLR, and LRP1. Moreover, the CCC complex regulates the phosphorylation and endosomal recruitment of the PtdIns(3P) phosphatase MTMR2 and then it primes membrane for the subsequent hydrolysis of PtdIns(3P). Therefore, the CCC complex–MTMR2 interaction is pivotal for activation of the WASH complex which drives the generation of branched F-actin filaments on endosomal membranes and thus the fission of recycling tubules [75,107,108,109,110,111].

The ability of RAB11 to set the time for PtdIns conversion through changes in its nucleotide binding status links RAB11 activation cycle with both endosome dynamics and membrane surface proteome remodeling. Therefore, perturbation of RAB11 activity might be a strategy to alter the biochemical composition of cellular membranes paving the way for fine control of complex cellular processes (Figure 1A).

In the sections below, we will discuss recent attempts aimed at supporting the therapeutic potential of RAB11 pharmacological targeting in human cancers.

## 7. RAB11 Targeting for Human Cancer Treatment

The significant role played by RAB11-mediated trafficking pathways in human cancers indicate that RAB11 isoforms are important targets for drug development. However, given the presence of conserved structural features shared by the over 60 members composing the RAB protein family, it makes it difficult to develop RAB11 selective inhibitors. Therefore, current pharmacological approaches are designed to block either the localization of RAB11 at membranes or the binding of specific effector molecules rather than to inhibit RAB11 nucleotide binding ability (Table 5). 

In particular, hypolipidemic agents such as Pitavastatin, Simvastatin, Fluvastatin block the generation of isoprenoid chains by 3-hydroxy-3-methyl-glutaryl-coenzyme A reductase (HMG-CoA reductase) and therefore both prenylation and membrane localization of Rab GTPase proteins including the RAB11 subfamily. Notably, both Pitavastatin and Simvastatin were found effective to reduce brain metastatic adaptation and outgrowth of breast cancer cells [43,114,115]. 

On the contrary, CDKI-73, a known cyclin-dependent kinase inhibitor, does not affect RAB11 membrane localization despite the fact that it was found to alter both endosome morphology and RAB11-mediated trafficking. Although the mechanism of action of this compound is currently unknown, it may be possible that CDKI-73 acts indirectly by perturbing endosome dynamics rather than activation/localization/effector binding of RAB11 isoforms [113].

Recently, a first generation of RAB11-mediated trafficking pathway inhibitors was developed targeting the RAB11:FIP binding interface, thereby enabling pharmacologic disruption of RAB25 and/or RAB11 signaling in cells. In particular, these inhibitors named RFP14, RFP24 and RFP26 were developed by engineering the RAB11 binding domain of RAB11FIP proteins and improving both their stability, cell-permeability and isoform selectivity (Table 5). Notably, these molecules were proven effective to block RAB11-mediated trafficking phenotypes in several cancer cell lines derived from breast and ovarian tissues [62]. 

## 8. Conclusions

RAB11 isoforms play crucial functions in cellular physiology by ensuring the maintenance of the biochemical composition of cellular membranes. A broad range of knowledge supports a role for these small GTPases in cancer progression. However, we are still struggling to understand the pathological implications of dysfunctional RAB11-mediated transport in tumors.

Novel ways to interpret RAB11 dysfunction comes from basic research studies. These reveal that proper RAB11 function requires—(i) assembly of large multiprotein complexes, (ii) cross-regulation between endosomal membrane domains with opposing functions, (iii) interlinked control of both RAB11 GTPase switch cycle and PtdIns(3)P metabolism.

We considered that research aimed at expanding and connecting these aspects will provide novel exciting mechanistic insights to understand how RAB11-mediated trafficking pathways sustain replenishment of the cell surface and hence they will offer novel insights into the role of RAB11 dysfunction in human cancers.

## Figures and Tables

**Table 1:** Structure of RAB11 gene isoforms.

Gene Name	Chromosome Position	Number of Coding Exon	Number of Amino Acids	Percentage of Identity	Tissue Expression
RAB11A	15q22.31	5	216	100% (with RAB11A)	Ubiquitous
RAB11B	19p13.2	5	218	89% (with RAB11A)	Wide, enriched in brain testis, heart
RAB25	1q22	5	213	61% and 66% (with RAB11A and RAB11B)	Wide, enriched in lung kidney, gastric tract

**Table 2:** Roles of RAB11A in cancer.

Roles	Expression Status	Cancer Type	Mechanism of Action	Function	References
Oncogenic	Over expression	Breast cancer	Increased signaling (ERK) Increased expression (EGFR)	Proliferation	[38]
		Non-small cell lung cancer	Decreased signaling (Hippo)	Tumorigenesis invasion	[39]
		Hepatocellular carcinoma	Increased signaling (PI3K/AKT) Increased expression (MMP2)	Tumorigenesis invasion	[40]
		Pancreatic cancer	Increased signaling (GSK3β/Wnt/β-catenin)	Proliferation invasion	[41]
Tumor suppressive	Under expression	Colon cancer	Decreased signaling (Hippo/YAP/IL6)	Tumorigenesis	[42]

**Table 3:** Roles of RAB11B in cancer.

Roles	Expression Status	Cancer Type	Mechanism of Action	Function	References
Oncogenic	Over expression	Breast cancer	Increased expression (β1-Integrin)	Metastasis	[43]
Tumor suppressive	Not yet defined	Not yet defined	Not yet defined	Not yet defined	Not yet defined

**Table 4:** Roles of RAB25 in cancer (adapted from [44]).

Roles	Expression Status	Cancer Type	Mechanism of Action	Function	References
Oncogenic	Over expression	Bladder cancer	Increased signaling (AKT/GSK3β /SNAIL)	Tumorigenesis metastasis	[45,46]
		Gastric cancer	Increased signaling (β1-Integrin/EGFR/SNAIL)	Invasion metastasis	[47,48]
		Glioblastoma	Increased signaling (AKT)	Tumorigenesis	[49]
		Liver cancer	Increased signaling (AKT/WNT)	Invasion	[50]
		Luminal Breast cancer	Increased signaling (β1-Integrin/EGFR/SNAIL)	Tumorigenesis metastasis	[48,51]
		Lung Cancer	Increased signaling (EGFR)	Tumorigenesis invasion	[52,53]
		Ovarian cancer	Increased signaling (β1-Integrin/EGFR/SNAIL)	Tumorigenesis metastasis	[48,51]
		Prostate cancer	Not yet defined	Tumorigenesis invasion	[54,55]
		Wilms	Not yet defined	Tumorigenesis	[56]
Tumor suppressive	Under expression	Colon cancer	Increased expression (EGFR)	Tumorigenesis	[32]
		Oesophageal cancer	Reduced signaling (ERK/FAK)	Tumorigenesis invasion	[57]
		Head and Neck cancer	Reduced cytoskeleton (F-actin)	Metastasis	[58]
		Triple negative breast cancer	Reduced expression (VEGF/VEGFR)	Tumorigenesis	[34,59]

**Table 5:** Inhibitors of RAB11-mediated trafficking pathways.

Molecule Name	Mechanism of Action	Specificity	Reference
Pitavastatin	HMG-CoA reductase inhibitor	Not yet determined	[43]
Simvastatin	HMG-CoA reductase inhibitor	Not yet determined	[43,112]
Fluvastatin	HMG-CoA reductase inhibitor	Not yet determined	[112]
CDKI-73	Not yet determined	Not yet determined	[113]
RFP14	RAB11:FIP interaction inhibitor	RAB25	[62]
RFP24	RAB11:FIP interaction inhibitor	RAB25	[62]
RFP26	RAB11:FIP interaction inhibitor	RAB11A, RAB25	[62]

## Data Availability

This study did not report any data.
